# Laparoscopic Management of Adhesions Developed after Peritoneal Nonclosure in Primary Cesarean Section Delivery

**DOI:** 10.1155/2018/6901764

**Published:** 2018-02-01

**Authors:** Emaduldin Seyam, Emad Moussa Ibrahim, Ayman Moheb Youseff, Eissa M. Khalifa, Enas Hefzy

**Affiliations:** ^1^Obstetrics and Gynecology Department, Minia University College of Medicine, Minya, Egypt; ^2^Microbiology and Immunology Department, Fayoum University College of Medicine, Faiyum, Egypt

## Abstract

**Objective:**

The aim of the current study was to laparoscopically investigate the effects of peritoneal nonclosure on the sites, types, and degrees of adhesions developed after primary caesarean section (CS) in women complaining of secondary infertility after first CS delivery.

**Study Design:**

This was a cross-sectional study, where 250 women suffering from secondary infertility after their first CS had been recruited. They had been classified into group I (*n* = 89), where both the visceral and parietal peritoneum had been left opened; group II (*n* = 75), where only the parietal peritoneum had been closed; and group III (*n* = 86), where both peritoneal layers had been closed. Laparoscopy had been used to classify those adhesions according to the location, severity, and their adverse impact on the reproductive capacity.

**Results:**

Both adnexal and nonadnexal adhesions had been found significantly higher in group I, while adnexal types of adhesions were significantly higher after nonclosure of the visceral peritoneum in group II. Laparoscopic tubal surgery performed included tubo-ovariolysis, fimbrioplasty, and neosalpingostomy. Pregnancy rate was found correlating with the adnexal adhesion location and score.

**Conclusion:**

Nonclosure of the peritoneum in CS is associated with more adhesion formation, which might adversely affect the future women reproduction.

## 1. Introduction

Cesarean section (CS) is the most common surgical procedure performed worldwide. The surgical approach of lower segment cesarean section has traditionally included closure of the visceral and the parietal peritoneum. The suggested advantages of closure of the peritoneum include restoring the normal anatomy and reapproximating the tissues, reducing the incidence of infection by reestablishing the natural anatomical barrier, reducing wound dehiscence, reducing hemorrhage, and minimizing the incidence of adhesion formation [1–3].

Adhesions developed after CS procedure are associated with multiple adverse effects, manifesting themselves as chronic pain, female delayed conception, and different degrees of bowel obstruction. Moreover, postoperative adhesions have been associated with prolonged delivery of infants during repeated CS. Peritoneal healing had been reported before to occur by metaplasia of the underlying connective tissue. Peritoneum regenerates within 5–8 days after surgery [4–7].

Compared to other laparotomies, peritoneal healing occurring after CS is slightly different because the 14- to 18-week-sized puerperal uterus in the midline would push the omentum and intestines through the peritoneal incision, delaying the natural peritoneal healing process and thus facilitating the adhesion formation in between the pushed organs, binding the anterior abdominal wall to uterus, omentum, and intestine as well [8–10].

Advantages suggested for peritoneal nonclosure include (1) reduction of operation duration, shortening of hospitalization, less analgesia used, earlier return of bowel function, and immediate postoperative recovery. It would also reduce the number of stitches which is a preferred option, as body responds to stitches as if they were a foreign material. Postoperative pain developed after peritoneal closure can cause unpleasant physiologic responses including retention of secretions in the respiratory system, ileus, and finally delayed breast-feeding [2, 5, 9].

The influence of traditional surgical closure of the peritoneum in CS on the development of adhesions is still unclear, especially if those adhesions could adversely affect the future reproduction after CS procedure. Most of the studies, investigating this issue, had conflicting results. Therefore, Cochrane reviewers determined that additional studies are required to determine the influence of closing the peritoneum on the long-term results of CS [10–13].

Fallopian tube abnormalities account for more than 40% of female subfertility and are mostly following laparotomies. Conventional surgical laparotomies often cause peritubal and perifimbrial adhesions, preserving the tubal mucosal integrity, and so laparoscopic adhesiolysis could successfully improve fecundability in 50% of cases, depending on the severity of tubal damage, associated abnormalities, techniques of laparoscopic surgery, and surgeons' experience [6, 9, 11, 14].

The aim of the current work is to laparoscopically assess the adhesions developed following first CS delivery as a result of peritoneal nonclosure during CS operation, in women suffering from secondary infertility, and evaluate the reproductive outcome after their management as well.

### 1.1. Patients and Methods

This cross-sectional study was conducted to clarify the long-term influence of peritoneal nonclosure, regarding the adhesion formation after first CS delivery. The study was performed in a University Hospital, which is a tertiary referral centre in Upper Egypt, after approval from the regional university ethical committee. The recruited women (*n* = 250), who had first CS delivery, had been recruited among more than 1500 cases who were candidates for laparoscopy for infertility causes, in the period between January 2012 and December 2016.

After revising the previous CS operative details of the women recruited (*n* = 250), categorization had been performed into group I (*n* = 89), where both the visceral and the parietal peritoneum had been left opened, nonclosed, group II (*n* = 75) where only the parietal peritoneum had been closed, leaving the visceral peritoneum opened, and group III (*n* = 86), where both the visceral and the parietal peritoneum had been closed.

None of the recruited women had a previous neither abdominal nor vaginal operation before or after the first CS delivery. Other causes of pelvic adhesions, such as pelvic inflammatory diseases (PID) or known cases of endometriosis, had been excluded. Women with medical complications, such as systemic lupus erythematosus (SLE), diabetes mellitus (DM), and idiopathic thrombocytopenic purpura (ITP), were also excluded from the study.

Conventional laparoscopy under general anesthesia using 10 mm umbilical and two 5 mm subsidiary trocars was performed with the same surgeon. Panoramic laparoscopic pelvic view shows the uterus, tubes, both ovaries, and uterovesical and Douglas pouches, in addition to both the parietal and the visceral peritoneum, to detect any abnormality concerning the size, shape, and mobility. Tubal patency has been confirmed using methylene blue dye test.

Adhesions detected during laparoscopy had been classified according to the locality into (1) adnexal adhesions, which were shown partially or totally covering the tube or ovary, or both; adnexal adhesions might interfere with the free fimbrio-ovarian movement or lead to fimbrial agglutination and fimbrial block, and (2) nonadnexal adhesions, which were shown between the uterus and bladder anteriorly, or the colon posteriorly, or fixing the fundus to the abdominal wall.

Adhesions shown had been classified according to the severity into the following types: (1) Mild adhesions, which are thin filmy avascular translucent or transparent adhesions and are easily cut with blunt dissection and subsequently easily freeing adherent organs. (2) Moderate adhesions, which are opaque, moderately thick layers, with moderate degree of vascularity and bleed minimally on dissection. (3) Extensive and severe adhesions, which are very thick, opaque, and mostly highly vascular and include omental and intestinal adhesions as well, and bleed much on dissection.

Evaluation of the relationship between the grades of adhesions detected, regarding the locality and severity and their possible adverse effects on the reproductive integrity of the internal genital organs, had been performed, either after peritoneal closure or left opened after their CS. All those adhesions had been successfully managed during the same laparoscopic session. Other abnormal pelvic findings had been diagnosed as well such as endometriosis and anomalies. Cumulative pregnancy rate (CPR) had been followed up for three to six months.

### 1.2. Statistics

The sample size was calculated based on the primary outcome measure that was the presence of adhesions. Assuming an adhesion rate of 60% in the nonclosure group, the sample size was calculated for a 50% reduction of the adhesion rate, with an alpha of 0.05 and a power of 80. Given this calculation, the minimum size was 45 cases in each group. The overall pregnancy rate (IUP and EP) was calculated taking into consideration only women followed up during the study period. The cumulative pregnancy rate was also calculated. For statistical analysis, Student's *t*-test, Mann–Whitney *U* test, Pearson's chi-square test, or Fisher's exact test was used, as appropriate; *P* < 0.05 was considered significant.

## 2. Results

Two hundred and fifty (*n* = 250) cases of laparoscopy were done at the Gynecological Endoscopic Surgery Day Surgery Unit for delayed conception after first CS delivery. The age of women recruited was between 19 and 39 years. The mean age of the patients was 25.6 ± 5.  Table 1 shows the characteristics of the study population. The women aged less than 35 years represented 88% of our sample, and the mean duration of infertility was 2.29 ± 5.9 years.

There was no remarkable difference between the groups regarding the age, BMI, and duration of infertility as mentioned in Table 1. All laparoscopic procedures had passed smoothly without intra- or postoperative complications. Only 10 women had to stay in the hospital under observation and to leave the hospital next day, but the rest of the women had left the hospital on the same day after few hours' admission after the laparoscopic procedures.

Both adnexal and nonadnexal adhesions were found in 97 and 88 cases, respectively. As shown in Table 2, group I had a significantly higher incidence of both types of adhesions, and more than half of those cases had moderate to severe degree of adhesions either adnexal or nonadnexal. This is in contrast to group III, where almost 85% of cases did not show any types of adhesions, and those that had adhesions were almost of mild degree of severity either adnexal or nonadnexal.

In group II, most adhesions found were of adnexal type with 43% of them of moderate and severe degree, in addition to another 15% of adhesions that could be seen between the uterus and the bladder; most of those were of mild degree of severity. Other types of adhesions were almost found in group I, which included omental and intestinal adhesions and those between the uterus and abdominal wall; most of those adhesions were dense, extensive, and highly vascular.

In cases where only the parietal peritoneum had been closed (group II), visceral peritoneal (adnexal) adhesions were the most common type of adhesions seen, which had significantly affected the tubal motility with different grades of adhesion severity. Those few cases in group (III), who showed adhesions, were almost very thin, filmy, and away from both the tubes. Other abnormal findings had been found during the laparoscopic sessions. Mild degree of pelvic endometriosis had been found in three cases in group III, two cases in group II, and one case in group I.

Table 3 shows the pregnancy rate after laparoscopic tuboplasty. Of the 97 women followed up after laparoscopic tuboplasty, 35 women (36%) got pregnant with 33 (94.2%) of them being intrauterine, while 2 (5.7%) of them were extrauterine pregnancies. Adhesion scoring regarding the site and severity were two important factors which could influence the occurrence of pregnancy after adhesiolysis procedure. The lowest degree of adnexal adhesions in group III was associated with the highest pregnancy rates (40.2%), while the lowest pregnancy rates (8.8%) was seen in group I having the highest degree of adnexal adhesions.

As shown in Table 3, the pregnancy outcome was significantly high after fimbrioplasty (fimbriolysis and fimbrio-ovariolysis) procedures more than those after proximal tubolysis and isolated ovariolysis, while it came lowest after salpingoneostomy procedure. Tubal pregnancy had been only seen after proximal tubolysis and salpingoneostomy procedures, while all pregnancies developed after the other laparoscopic tuboplasty procedures were intrauterine ones. Other types of adhesions had been successfully attacked laparoscopically with good haemostasis.

## 3. Discussion

Adhesion is one of the most important postoperative complications seen after laparotomies. Adhesion development after CS procedure would increase the subsequent CS operative time; increase the incidence of accidental traumas to the intestines, bladder, and ureter; and increase bleeding complications as well. It has been previously reported that between 6 and 8% of women who underwent cesarean section were readmitted to theatre for the management of adhesion complications developed after previous CS. So, adhesions could be considered as one of the important causative factors in secondary female infertility [15–18].

In the current study, closure of both the visceral and peritoneal layers in group III in previous CS procedure had shown the least degree of adhesions development, concerning both the site and degree of adhesions severity. This is in contrast to those cases in group I, where both the peritoneal layers had been left opened; those had been complicated with the highest degrees of adhesions, regarding both the site and the degree of adhesions severity as well. Adnexal type of adhesions was the one significantly affecting tubal motility, and so their successful laparoscopic management had shown the highest pregnancy outcome.

Controversies still exist regarding adhesion formation after peritoneal closure or leaving it opened in previous CS. Both opinions have their reasonable justifications. Opinion supporting nonclosure reported that peritoneal healing occurs by simultaneous multisite healing as the result of mesothelial cells migration with mesothelial matrix formation without the need for peritoneal reposition. They also added that peritoneal closure would lead to foreign body reactions to the suture material, ischemia, and tissue necrosis, interfering with natural healing in addition to increasing the incidence of adhesions formation [11, 14, 16–21].

According to the previously mentioned pathophysiological hypothesis, nonclosure of both the peritoneal layers would show less adhesion development. The second opinion reported that the uterus might take 6 weeks to return to its normal size and position, while peritoneal healing would occur within 3–5 days after CS. So, the enlarged postpartum uterus might act as a mechanical barrier against the routine natural mesothelial matrix formation and subsequent peritoneal healing, and this would be the cause of nonadnexal adhesions development between the uterus, omentum, intestine, and anterior abdominal wall, which is almost an extensive type of adhesion [22–26].

In the current study, in group II, the visceral peritoneum had been left opened, which had been complicating significant adhesions: both adnexal interfering with tubal motility and nonadnexal between the uterus and bladder, while closing the parietal peritoneum had not been complicated with adhesions, which could support our upcoming results. The procedure of closing the parietal peritoneum, leaving the visceral one opened is a preferred procedure with several surgeons—taking the benefits of leaving the visceral layer opened such as shortening the procedure time and avoiding bladder suspension and the benefits of closing the parietal peritoneum such as keeping the intestine and omentum in and facilitating the anatomical restoration during abdominal closure.

Recently, a meta-analysis compared adhesions after closure or nonclosure of the peritoneum during CS based on three well-organized RCTs; the authors of this meta-analytic work concluded that closure of the peritoneum had the advantage of significant reduction of adhesion formation. As CS procedure has a lot of technique modifications, it is not sufficient to differentiate groups regarding the adhesion formation only by closure and nonclosure of the peritoneum, because other variables like longer abdominal operation time or pushing down of the bladder might also contribute to adhesion development after previous laparotomies [11, 27–30].

Adhesion formation might also be related to suture material, tissue devascularization, ischemia, infection, amount of manipulation, and the degree of aseptic technique. Peritoneal healing differs from other epithelial tissues healing, where during the peritoneal healing process, the reepithelialization of peritoneal surfaces occurs simultaneously throughout the surgical site, as mesothelial cells migrate into the supportive matrix and would initiate multiple sites of healing. Mesothelial matrix formed would cover the peritoneal defects within approximately 3 days and is almost completed within 5–8 days, regardless of approximation of peritoneal ends [31–36].

It had been previously reported that the incidence of intra-abdominal adhesions in women who underwent repeated cesarean section delivery steadily rises with each subsequent CS delivery (24.4% after 2 cesarean deliveries, 42.8% after 3 cesarean deliveries, and 47.9% after 4 cesarean deliveries). However, the proportion of adhesion sites to the number of patients and the proportion of dense adhesion sites increased steadily across the second, third, and fourth cesarean deliveries, which could predict more adhesion sites and more dense adhesion after each subsequent cesarean delivery [37–39].

Peritoneal nonclosure in cesarean sections will certainly reduce the surgical procedure time by few minutes which had encouraged many previous studies to recommend peritoneal nonclosure, especially in CS deliveries. Moreover, another study conducted in a Military hospital in Pakistan, which had compared peritoneal closure versus nonclosure, observed that peritoneal nonclosure was recommended as it reduces the surgical procedure time and decreases anesthesia duration and medications, in addition to the quicker recovery and the early hospital discharge [32].

Successful laparoscopic management of adnexal adhesions in the current work had been positively correlated with subsequent higher cumulative pregnancy rate, especially in those women with less intense degree of adhesions severity. Tubo-ovarian movement is important for ovum pick-up, and so laparoscopic fimbriolysis and fimbrio-ovariolysis had shown the highest pregnancy outcome, while salpingoneostomy had shown significantly lower pregnancy outcome, as fimbrial amalgamation and block would not be improved after simple neosalpingostomy.

Moreover, the only 2 ectopic pregnancies developed after laparoscopic adhesiolysis were after both proximal tubolysis and salpingoneostomy, as the first procedure is associated with the normal tubal peristaltic movements and the second is associated with impaired normal cilia cells of the tubal mucosa, and both would increase the incidence of ectopic pregnancy. Adhesiolysis of nonadnexal adhesions had not improved the postoperative pregnancy outcome but had the benefits of freeing the intestine, omentum, and uterus as well.

Most studies which had recommended nonclosure of the peritoneum during CS procedure have never weighted the long-term adverse effects and the expected complications after successive surgical procedures including adhesion formation. A previously published double-blind randomized trial, which had been performed to compare the intensity of postcesarean pain between peritoneal closure and nonclosure group, had concluded that there is no difference in postoperative pain in both groups in successive cesareans sections [21, 32, 36, 38, 40–44].

Tulandi et al., in a review of 14 studies observed that nonclosure of the peritoneum had resulted in a significantly higher incidence of adhesion formation, which is going with our study results [3]. In another prospective randomized trial done by Zareian et al. which included 45 women, it reported longer operative time after peritoneal closure but positively found decreased risk of adhesions formation and thus suggested peritoneal closure during CS delivery [19]. Peritoneal closure is simply restoring the anatomical continuation of tissues already cut during CS procedure and thus restoring the normal function and integrity of the internal abdominal organs.

Before starting this work, our university department had made a questionnaire regarding peritoneal closure technique among more than 100 obstetrician and gynecologist. Seventy-five percent of sharers in this questionnaire confirmed their movement back to close the peritoneum during CS procedure after leaving it opened for many years before. Their change of mind decision is made on the significant adhesions found after leaving the peritoneum opened in the first CS delivery. Proper surgical techniques performed in the first CS delivery would definitely allow for easy, simple, and clean subsequent repeated CS procedures with the least degree of morbidity.

In conclusion, meticulous peritoneal closure under proper surgical handling of tissues, good haemostasis, and clean aseptic operative field would certainly lead to restoration of the healthy normal pelvic anatomy after CS procedure. We could also confirm that successive laparotomies after the first CS delivery would be cleaner from adhesions after peritoneal closure. So, to reduce adhesion development and other relevant reproductive morbidity, routine closure of both peritoneal layers under proper surgical techniques in cesarean sections is recommended.

## Figures and Tables

**Table 1:** Characteristics of women recruited for the study.

	Group I (*N* = 89)	Group II (*N* = 75)	Group III (*N* = 86)	*P*
Mean age (years)	29.4 ± 5.6	29.2 ± 5.1	29.3 ± 5.2	0.68
Mean BMI	25 ± 1.2	25 ± 2.3	25 ± 1.4	0.43
Mean infertility duration (years)	2.1 ± 0.6	2.2 ± 0.7	2.1 ± 0.5	0.65

**Table 2:** Adhesion formation difference in all groups.

	Group I (*N* = 89)	Group II (*N* = 75)	Group III (*N* = 86)	*P*
Adnexal				
None	34	40	79 (91.8%)	<0.001
Mild	5 (5.6%)	2 (2.7%)	5 (5.6%)	
Moderate	35 (39.3%)	23 (30.7%)	2 (2.3%)	
Severe	15 (16.8%)	10 (13.3%)	None	
Abdominal wall				
None	33	68 (90.7%)	80 (93%)	<0.01
Mild	8 (8.9%)	7 (9.3%)	4 (4.7%)	
Moderate	36 (40.4%)	3 (4%)	2 (2.3%)	
Severe	12 (13.4%)	None	None	
Uterus to bladder				
None	39	44	73 (85.9%)	<0.001
Mild	4 (4.5%)	5 (6.7%)	8 (9.3%)	
Moderate	36 (40.4%)	23 (30.7%)	5 (5.8%)	
Severe	10 (11.2%)	3 (4%)	None	
Uterus to abdominal wall				
None	35	68 (90.7%)	80 (93%)	<0.001
Mild	5 (5.6%)	4 (5.3%)	4 (4.7%)	
Moderate	34 (38.2%)	3 (4%)	2 (2.3%)	
Severe	15 (16.8%)	None	None	
Others				
None	54	67 (75.3%)	82 (95.3%)	<0.001
Mild	3 (3.4%)	5 (6.7%)	3 (3.4%)	
Moderate	25 (28%)	3 (4%)	1 (1.2%)	
Severe	7 (7.9%)	None	None	

**Table 3:** 

	Number of patients	Overall pregnancy, *n* (%)	IUP, *n* (%)	EP, *n* (%)	*P*
*Duration of infertility*					
1–3 years	57	20 (20.6%)	20 (57.1%)	None	0.05
3–5 years	40	15 (15.5%)	13 (37.1%)	2 (5.7%)	
*Type of tuboplasty*					
Fimbriolysis	35	12 (12.4%)	12 (34.3%)	None	0.03
Ovariolysis	18	8 (8.2%)	8 (22.9%)	None	
Fimbrio-ovariolysis	21	7 (7.2%)	7 (20%)	None	
Proximal tubolysis	15	5 (5.2%)	1 (14.3%)	1 (2.9%)	
Neosalpingostomy	8	3 (3%)	1 (5.6%)	1 (2.9%)	
*Adnexal adhesion*					
Mild	35	20 (20.6%)	20 (57.1%)	None	0.01
Moderate	44	10 (10.3%)	10 (28.6%)	None	
Severe	18	5 (5.2%)	3 (8.6%)	2 (5.7%)	

## Abbreviations

CS: Cesarean section
PID: Pelvic inflammatory diseases
SLE: Systemic lupus erythematosus
DM: Diabetes mellitus
ITP: Idiopathic thrombocytopenic purpura
CRP: Cumulative pregnancy rate
IUP: Intrauterine pregnancy
EP: Ectopic pregnancy.

## Data Access

The datasets used and/or analyzed during the current study are available from the corresponding author on reasonable request.
